# The risk of breast and gynecological cancer in women with a diagnosis of infertility: a nationwide population-based study

**DOI:** 10.1007/s10654-018-0474-9

**Published:** 2019-01-09

**Authors:** Frida E. Lundberg, Anastasia N. Iliadou, Kenny Rodriguez-Wallberg, Kristina Gemzell-Danielsson, Anna L. V. Johansson

**Affiliations:** 1 Department of Medical Epidemiology and Biostatistics, Karolinska Institutet, Box 281, 171 77 Stockholm, Sweden; 2 Department of Oncology-Pathology, Karolinska Institutet and Department of Gynecology and Reproduction, Section of Reproductive Medicine, Karolinska University Hospital, Stockholm, Sweden; 3 Department of Women’s and Children’s Health, Division of Obstetrics and Gynecology, Karolinska Institutet and Karolinska University Hospital, WHO Collaborating Centre, Stockholm, Sweden; 4 Cancer Registry of Norway, Oslo, Norway

**Keywords:** Infertility, Endometriosis, Anovulation, Polycystic ovary syndrome, Cancer

## Abstract

Some studies have suggested that infertility is a risk factor for endometrial, ovarian and breast cancer. The study aimed to create a comprehensive picture of the association between infertility and the risk of ovarian, endometrial and breast cancer, and whether any association could be explained by ovulatory disturbances, endometriosis or nulliparity. In a population-based cohort of 2,882,847 women, cox regression analysis was used to investigate cancer incidence among infertile women. Overall, infertility was associated with a higher incidence rate of ovarian (adjusted hazard ratio [aHR] 1.53, 95% confidence interval [CI] 1.38–1.71) and endometrial cancer (aHR 1.25, 95% CI 1.11–1.40), but not of breast cancer (aHR 0.96, 95% CI 0.92–1.01). Ovarian cancer incidence was higher in women diagnosed with endometriosis, and in nulliparous women with ovulatory disturbances, compared to women with none of the diagnoses. Endometrial cancer incidence was higher in women with ovulatory disturbances, but not in women with endometriosis. These findings suggest that infertility could have long-term consequences of importance to physicians and public health workers.

**Electronic supplementary material:**

The online version of this article (10.1007/s10654-018-0474-9) contains supplementary material, which is available to authorized users.

## Introduction

Infertility is among the most common diseases affecting young adults. It is estimated that one in seven couples trying to conceive are diagnosed with infertility [1], and female causes likely contribute to more than half of cases [2, 3]. Hormonal disorders affecting ovulation, including polycystic ovary syndrome (PCOS), are the most common causes of female infertility. Tubal factor infertility is also common, while endometriosis contributes to a smaller number of cases [2].

Cancers of the ovary, endometrium and breast are related to several hormonal and reproductive risk factors. Nulliparity, early menarche and late menopause are associated with higher risk of these malignancies, while the risk declines with each live birth [4–6]. The risk of breast cancer also increases with higher age at first birth [4], whereas pregnancy later in life seems to lower the risk of ovarian and endometrial cancer [5, 6]. Similarly, oral contraceptive use has been linked to a temporary increase in breast cancer risk [4], and a lower risk of ovarian and endometrial cancer [5, 6].

A number of previous studies have investigated the associations between fertility treatments using hormonal stimulation and the risk of breast and gynecological cancers. A recurring issue in these studies is the potential confounding by the underlying infertility [7–9]. Since both causes and consequences of infertility could influence cancer risk, the relationship is complex. The associations between endometriosis and ovarian cancer, as well as between PCOS and endometrial cancer, are fairly well established [10, 11]. Sustained estrogen stimulation to the endometrium without an opposed antiestrogenic effect of progesterone, which is the usual feature of chronic anovulation and PCOS, has a recognized carcinogenic effect in the development of some endometrial cancers. The association between endometriosis and ovarian cancer is less understood and inflammatory features associated with endometriosis have been proposed as predisposing to ovarian cancer [12]. Additionally, some studies have suggested that infertility per se is a risk factor for endometrial, ovarian and breast cancer [13–17], though this is debated [18–20]. It is also unclear whether these associations are attributable to any specific underlying cause of infertility.

The aim of this study was to create a comprehensive picture of the association between infertility and the risk of ovarian, endometrial and breast cancer in a large population-based cohort of Swedish women. We also aimed to investigate if such associations could be explained by either ovulatory disturbances, endometriosis or nulliparity.

## Materials and methods

### Study population

The study population comprised women born 1942–1992 who were registered in the population-based Swedish Multi-Generation Register (MGR) (n = 3,626,265). The MGR includes individuals born since 1932 and residing in Sweden 1961 or later, with links to their parents [21]. We used the personal identification number, which is uniquely assigned to all Swedish residents, for linkage between the MGR and other Swedish registers.

Women with invalid personal identification numbers (n = 4868) were excluded. Furthermore, as inclusion criteria women had to be alive and residing in Sweden at age 20 with less than five children. Hence, we excluded women who died prior to age 20 (n = 32,707), who immigrated after age 20 (n = 701,160), and women with more than four children prior to age 20 (n = 12). In addition, women diagnosed with any malignant disease before age 20 were excluded (n = 4671). After these criteria were applied, the cohort comprised 2,882,847 women. In all regression analyses, women missing information on education and/or country of birth were also excluded (n = 111,826). In the analyses of ovarian cancer incidence, 78 women who had bilateral oophorectomy before age 20 were also excluded. In the analyses of endometrial cancer incidence, 77 women with hysterectomy before age 20 were excluded.

### Exposure information

Information on diagnoses related to infertility was obtained from the National Patient Register (NPR), where in-patient care has been recorded since 1964 and specialist out-patient care since 2001, according to the current version of the International Classification of Diseases (ICD) [22]. The corresponding ICD codes for these diagnoses during the study period are listed in Supplementary Table 1.

To address the underlying cause of infertility, we used diagnoses of endometriosis and ovulatory disturbances in combination with infertility as a proxy for infertility due to these disorders. Prior to 2001, diagnoses of endometriosis and ovulatory disturbances were only recorded in in-patient records, and thus women with these diagnoses in out-patient care prior to 2001 will not be captured by the proxy. Ovulatory disturbances were defined as having a diagnosis of ovarian dysfunction, (including PCOS), menstrual disturbances (including absent, rare or scanty menstruation) or infertility due to anovulation. Women diagnosed with endometriosis or infertility due to endometriosis were categorized as having endometriosis.

We included diagnoses of female infertility, ovulatory disturbances and endometriosis at age 15 or later. Median ages at diagnosis were 30.6 years for infertility (inter-quartile range [IQR] 27.2–34.2), 27.4 years for ovulatory disturbances (IQR 22.4–33.3) and 35.4 years for endometriosis (IQR 28.4–43.7).

### Cancer

Since 1958, diagnoses of cancer in all Swedish residents have been recorded in the Swedish Cancer Register (SCR). The register includes date of diagnosis, hospital and tumor site coded using the 7th and current version of ICD.

In the present study, the cancers of interest were defined as morphologically verified malignant tumors of the breast (ICD-7 code 170), ovary, fallopian tube and broad ligaments (ICD-7 175), or uterine body (ICD-7 172). Women with a first diagnosis of these cancers during follow-up were considered cases. Women with any diagnosis of malignant disease (ICD-7 140-205) before start of follow-up were excluded, and women diagnosed with a malignant disease other than those studied during the study period were censored at date of diagnosis. The 83 women who were diagnosed with both ovarian and endometrial primary malignant tumors on the same date were included as cases for both cancer types.

### Other covariates

The woman’s date of birth, parity and birthdate of each child were obtained from the MGR.

From the Total Population Register we obtained each woman’s country of birth as well as any migrations in or out of Sweden. Date of death was obtained from the Cause of Death Register and highest attained education level from the Education Register.

The NPR was used to identify women who had gone through bilateral oophorectomy, hysterectomy and salpingectomy. Surgeries performed up to 60 days before cancer diagnosis were assumed to be related to the cancer treatment and not included as covariates or censoring events (483 bilateral oophorectomies, 498 hysterectomies and 634 salpingectomies).

### Statistical analyses

Person-time at risk was accrued from the date of the 20th birthday until the date of first breast or gynecological cancer diagnosis, or censoring at date of other cancer diagnosis, death, emigration or birth of the fifth child. Censoring at fifth child was done to simplify analyses, since follow-up time was split on parity and only 1% of cancer cases occurred in women with five or more children. In the analysis of ovarian cancer incidence, follow-up time was also censored at date of bilateral oophorectomy, while date of hysterectomy was used for censoring in analyses of endometrial cancer, since these women were no longer at risk for each respective tumor.

Cox proportional hazard models were used to estimate hazard ratios (HRs, which can be interpreted as incidence rate ratios), with 95% confidence intervals (CIs) for the cancers of interest, using attained age as the timescale.

In the main models, infertility was entered as a time-dependent exposure at the date of diagnosis. Women with and without infertility diagnosis were subdivided into three groups having ovulatory disturbances, endometriosis, or neither, resulting in six exposure categories. These categories were entered into the models as time-dependent exposures, i.e. a woman changed exposure category at the earliest date of diagnosis and could therefore contribute person-time to more than one category. Women diagnosed with both ovulatory disturbances and endometriosis were included in the category ovulatory disturbances (n = 2356).

Calendar time (split into 10-year intervals), parity/age at first birth and salpingectomy were included in the models as time-dependent covariates. When not used for censoring, hysterectomy and bilateral oophorectomy were also included as time-dependent covariates. Country of birth (Nordic, non-Nordic) and education level (9-year compulsory, secondary school < 3 or 3 years, higher education < 3 or ≥ 3 years) were included as fixed covariates. Current parity (nulliparous; 1, 2, 3 or 4 children) and age at first birth (< 25, 25–35 or ≥ 35 years) were combined into 13 categories.

The proportional hazards assumption was assessed using tests based on Schoenfeld residuals. The hazards were proportional overall, with some indication of stronger relative effects on ovarian cancer in younger ages where rates (and number of events) were low. Since early and late onset cancers may have different risk factors, we investigated potential differences between pre- and postmenopausal cancer by splitting follow-up time at the 50th birthday (as an approximation of menopause). For each outcome, we also examined interactions with nulliparity, through splitting follow-up time at the date of first birth. Interactions were assessed using likelihood ratio tests comparing models with and without interaction terms. In sensitivity analyses, women diagnosed with both ovulatory disturbances and endometriosis were excluded from the main models, to investigate whether any associations with ovulatory disturbances were influenced by co-occurring endometriosis. For each outcome, multivariable adjusted models not including parity/age at first birth were fitted to examine potential mediation through these factors.

Data was prepared using SAS software (version 9.4, SAS Institute Inc., Cary, N.C., USA) and statistical analyses performed using Stata (StataCorp. 2017. *Stata Statistical Software: Release 15.* College Station, TX: StataCorp LLC). All tests were two-sided with a significance level of 5%.

The Ethical Review Board in Stockholm, Sweden approved this study (ethical approval 2013/1849-31/2, amendment 2014/118-32).

## Results

Study population characteristics, by diagnosis of infertility, are presented in Table 1. Women born before 1950 and after 1980 were less likely to have a diagnosis of infertility. Infertile women were more likely to have higher education, to be older at their first birth and to have fewer children at the end of the study follow-up. Among infertile women, 19,299 (16.4%) were diagnosed with ovulatory disturbances and 14,030 (11.9%) with endometriosis, compared to 41,617 (1.5%) and 54,797 (2.0%) women with no infertility diagnosis. A history of salpingectomy or hysterectomy was more common among infertile women.

**Table 1:** Characteristics of the study population by diagnosis of infertility

Characteristic	Not infertile (n = 2,765,347)		Infertile (n = 117,500)	
*Birthyear*	N	%	N	%
1942–1949	479,293	17.3	13,704	11.7
1950–1959	514,247	18.6	25,747	21.9
1960–1969	541,828	19.6	25,858	22.0
1970–1979	501,622	18.1	37,080	31.6
1980–1992	728,357	26.3	15,111	12.9
*Highest education level*				
9-year compulsory	391,864	14.2	11,625	9.9
Secondary school 1–2 years	632,388	22.9	28,726	24.4
Secondary school 3 years	627,863	22.7	23,930	20.4
Higher education < 3 years	403,416	14.6	17,128	14.6
Higher education ≥ 3 years	598,666	21.6	35,415	30.1
Missing	111,150	4.0	676	0.6
*Country of birth*				
Nordic	2,591,062	93.7	111,240	94.7
Non-Nordic	172,888	6.3	6260	5.3
Missing	1397	0.1	0	0.0
*Ovulatory disturbances, ever*	41,617	1.5	19,299	16.4
*Endometriosis, ever*	54,797	2.0	14,030	11.9
*Age at first birth*				
Nulliparous	902,934	32.7	36,589	31.1
< 25 years	914,220	33.1	19,852	16.9
25–34 years	871,638	31.5	45,914	39.1
≥ 35 years	76,555	2.8	15,145	12.9
*Parity at end of follow*-*up*				
Nulliparous	902,934	32.7	36,589	31.1
One child	392,630	14.2	33,638	28.6
Two children	925,135	33.5	33,028	28.1
Three children	405,158	14.7	11,038	9.4
Four children	139,490	5.0	3207	2.7
*Salpingectomy*	77,746	2.8	12,311	10.5
*Hysterectomy*	103,909	3.8	6377	5.4
*Bilateral oophorectomy*	22,638	0.8	1974	1.7

Of the 2,882,847 women in the cohort, 117,500 (4.1%) had a diagnosis of infertility. The mean follow-up time was 23.4 years among non-infertile women and 25.8 years among women with infertility. In non-infertile women, 52,601 women were diagnosed with breast cancer, 6337 with ovarian cancer and 6152 with endometrial cancer during 64,765,032 years of follow-up. Among women with a diagnosis of infertility, there were 2133 breast cancer, 392 ovarian cancer and 318 endometrial cancer cases, respectively, during 3,035,653 person-years. Among non-infertile women, the crude incidence rates were 81.2 cases of breast cancer, 9.8 cases of ovarian cancer and 9.0 cases of endometrial cancer per 100,000 person-years. The corresponding rates among infertile women, were 70.3 breast cancer cases, 12.9 ovarian cancer cases and 10.5 endometrial cancer cases per 100,000 person-years, respectively.

### Breast cancer

Results for breast cancer incidence are presented in Table 2. Neither infertility nor related diagnoses were associated with a higher incidence of breast cancer in the multivariable adjusted analysis. Parity and age at first birth did not seem to influence these results (Supplementary Table 2).

**Table 2:** Associations between infertility and breast cancer incidence

	Cancer cases	Person-years	Age-adjusted		Multivariable^a^	
			HR (95% CI)	*p* value	HR (95% CI)	*p* value
*Infertility*						
No infertility	51,575	64,702,934	1.00 (reference)	.	1.00 (reference)	.
Infertility	2112	1,759,886	1.05 (1.00–1.10)	0.029	0.96 (0.92–1.01)	0.111
*Infertility and related diagnoses*						
No diagnosis	49,822	63,500,014	1.00 (reference)	.	1.00 (reference)	.
Ovulatory disturbances	330	374,986	0.97 (0.87–1.08)	0.589	0.94 (0.84–1.05)	0.274
Endometriosis	1423	827,934	1.01 (0.95–1.06)	0.845	1.02 (0.96–1.07)	0.569
Infertility	1733	1,404,738	1.06 (1.01–1.11)	0.024	0.98 (0.93–1.02)	0.309
Infertility and ovulatory disturbances	114	153,635	0.99 (0.82–1.18)	0.874	0.89 (0.74–1.07)	0.206
Infertility and endometriosis	265	201,513	1.03 (0.92–1.17)	0.592	0.93 (0.83–1.06)	0.275

*p* < 0.05 is significant in all analyses. The dots represent the reference category

^a^Adjusted for age, calendar time, education level, country of birth, parity and age at first birth, salpingectomy, hysterectomy and bilateral oophorectomy

We found no evidence for an effect modification by parity (adjusted *p* value = 0.2580). These results are presented in Supplementary Table 3.

When stratifying by age, the effect modification was statistically significant (adjusted *p* value 0.0124) (Supplementary Table 3). While none of the diagnoses were associated with post-menopausal breast cancer incidence, the incidence of pre-menopausal breast cancer was somewhat lower among women diagnosed with infertility and endometriosis (adjusted HR [aHR] = 0.79 [95% CI 0.65–0.95]), or infertility alone (aHR 0.92, 95% CI 0.86–0.99).

### Ovarian cancer

Among all infertile women, the adjusted HR of ovarian cancer was 1.53 (95% CI 1.38–1.71) compared to women without infertility (Table 3). The incidence of ovarian cancer was higher among women with ovulatory disturbances (aHR 1.53, 95% CI 1.18–1.98), endometriosis (aHR 1.77, 95% CI 1.53–2.05) and infertility (aHR 1.53, 95% CI 1.36–1.72), compared to women with none of these diagnoses. Women diagnosed with both infertility and ovulatory disturbances did not have a significantly higher incidence of ovarian cancer (aHR 1.12, 95% CI 0.68–1.87), though the small number of cases in this group produced uncertain estimates. The highest incidence of ovarian cancer was seen in women diagnosed with both infertility and endometriosis (aHR 2.19, 95% CI 1.70–2.82). The associations between infertility, related diagnoses and ovarian cancer were slightly stronger when not adjusting for parity and age at first birth (Supplementary Table 2).

**Table 3:** Associations between infertility and ovarian cancer incidence

	Cancer cases	Person-years	Age-adjusted		Multivariable^a^	
			HR (95% CI)	*p* value	HR (95% CI)	*p* value
*Infertility*						
No infertility	6060	64,479,845	1.00 (reference)	.	1.00 (reference)	.
Infertility	375	1,739,692	1.73 (1.56–1.92)	< 0.001	1.53 (1.38–1.71)	< 0.001
*Infertility and related diagnoses*						
No diagnosis	5793	63,340,911	1.00 (reference)	.	1.00 (reference)	.
Ovulatory disturbances	58	371,256	1.53 (1.18–1.98)	0.001	1.53 (1.18–1.98)	0.001
Endometriosis	209	767,678	1.55 (1.35–1.78)	< 0.001	1.77 (1.53–2.05)	< 0.001
Infertility	299	1,393,212	1.71 (1.52–1.92)	< 0.001	1.53 (1.36–1.72)	< 0.001
Infertility and ovulatory disturbances	15	152,397	1.15 (0.69–1.91)	0.589	1.12 (0.68–1.87)	0.651
Infertility and endometriosis	61	194,084	2.38 (1.85–3.06)	< 0.001	2.19 (1.70–2.82)	< 0.001

*p* < 0.05 is significant in all analyses. The dots represent the reference category

^a^Adjusted for age, calendar time, education level, country of birth, parity and age at first birth, salpingectomy and hysterectomy

Results from the models stratified by parity and by age are presented in Supplementary Table 4. The effect modification was statistically significant (adjusted *p* value = 0.0333). Among nulliparous women, diagnoses of ovulatory disturbances, endometriosis or infertility were all associated with a higher incidence of ovarian cancer, compared to women with none of these diagnoses. Parous women diagnosed with endometriosis, infertility or a combination of both also had a higher incidence of ovarian cancer. Ovulatory disturbances, with or without infertility, were not significantly associated with an increased incidence of ovarian cancer in parous women.

The associations with ovarian cancer were modified by age (adjusted *p* value = 0.0002) (Supplementary Table 4). Compared to women with no infertility-related diagnosis, women with ovulatory disturbances had a higher incidence of ovarian cancer before menopause, but not after. The incidence of both pre- and post-menopausal ovarian cancer was higher in women diagnosed with endometriosis, infertility, or both. These associations were stronger before menopause. Women diagnosed with ovulatory disturbances and infertility did not have significantly higher incidence of either pre- or postmenopausal ovarian cancer.

### Endometrial cancer

Infertile women had a higher incidence of endometrial cancer (aHR 1.25, 95% CI 1.11–1.40) compared to fertile women (Table 4). Women diagnosed with either infertility (aHR 1.21, 95% CI 1.07–1.38) or ovulatory disturbances (aHR 1.45, 95% CI 1.12–1.88) had a higher incidence of endometrial cancer, and the highest incidence was found in women with both these diagnoses (aHR 2.90, 95% CI 2.05–4.08). However, the incidence of endometrial cancer was not increased among women exclusively diagnosed with endometriosis (aHR 0.94, 95% CI 0.76–1.17), or in combination with infertility (aHR 0.87, 95% CI 0.58–1.30). The associations with infertility and ovulatory disturbances were stronger when not adjusting for parity and age at first birth (Supplementary Table 2).

**Table 4:** Associations between infertility and endometrial cancer incidence

	Cancer cases	Person-years	Age-adjusted		Multivariable^a^	
			HR (95% CI)	*p* value	HR (95% CI)	*p* value
*Infertility*						
No infertility	5797	63,265,993	1.00 (reference)	.	1.00 (reference)	.
Infertility	315	1,676,408	1.55 (1.39–1.74)	< 0.001	1.25 (1.11–1.40)	< 0.001
*Infertility and related diagnoses*						
No diagnosis	5651	62,380,847	1.00 (reference)	.	1.00 (reference)	.
Ovulatory disturbances	57	363,077	1.52 (1.17–1.97)	0.002	1.45 (1.12–1.88)	0.005
Endometriosis	89	522,069	1.00 (0.81–1.24)	0.985	0.94 (0.76–1.17)	0.584
Infertility	258	1,351,728	1.51 (1.33–1.71)	< 0.001	1.21 (1.07–1.38)	0.003
Infertility and ovulatory disturbances	33	149,925	3.33 (2.37–4.70)	< 0.001	2.90 (2.05–4.08)	< 0.001
Infertility and endometriosis	24	174,755	1.14 (0.76–1.70)	0.534	0.87 (0.58–1.30)	0.503

*p* < 0.05 is significant in all analyses. The dots represent the reference category

^a^Adjusted for age, calendar time, education level, country of birth, parity and age at first birth, salpingectomy and bilateral oophorectomy

The associations between ovulatory disturbances, infertility, and endometrial cancer appeared to be stronger in nulliparous women (adjusted *p* value = 0.0414) (Supplementary Table 5). Being diagnosed with both infertility and ovulatory disturbances was associated with a higher incidence of endometrial cancer in both nulliparous (aHR 3.46, 95% CI 2.22–5.38) and parous women (aHR 2.28, 95% CI 1.32–3.93). Nulliparous women diagnosed with either ovulatory disturbances (aHR 2.20, 95% CI 1.51–3.20) or infertility (1.28, 95% CI 1.08–1.53) also had increased incidences of endometrial cancer (Table 4).

When stratifying by age, the higher incidence of endometrial cancer was only present in women below the age of 50 (adjusted *p* value < 0.0001) (Supplementary Table 5). After adjustments, the incidence of pre-menopausal endometrial cancer was higher in women diagnosed with ovulatory disturbances (aHR 4.09, 95% CI 2.79–5.99), infertility (aHR 1.66, 95% CI 1.29–2.13), or both (aHR 8.09, 95% CI 5.38–12.15). None of the diagnoses were associated with a significantly higher incidence of post-menopausal endometrial cancer.

### Sensitivity analysis

Excluding the 2356 women diagnosed with both ovulatory disturbances and endometriosis for the analyses of breast cancer, ovarian cancer and endometrial cancer did not alter the interpretation of the results in any of the analyses.

## Discussion

In this large population-based study, we examined the risk of cancer in women with a diagnosis of infertility, and examined explanatory variables such as ovulatory disturbances, and endometriosis as well. Our overall findings indicate that infertile women had higher risks of ovarian and endometrial cancer, but the risk of breast cancer was not found to be increased. Ovulatory disturbances were associated with higher ovarian cancer risk among nulliparous women, and with higher endometrial cancer risk overall. Endometriosis was associated with a higher risk of ovarian cancer but not of endometrial cancer.

### Breast cancer

We found no increase in breast cancer risk among women with infertility or related diagnoses. These findings are in line with several previous studies on breast cancer risk in women with infertility [23–25], PCOS, [26], or endometriosis [27]. However, in a recent study, Jensen et al. [14] found a slightly higher risk of breast cancer in a cohort of infertile women, compared to the general population. The reported standardized incidence ratios (SIRs) were based on population rates adjusted for parity, age and calendar time. In our study, infertility was associated with a higher risk of breast cancer in the age-adjusted models, but not in the fully adjusted analysis. It is possible that the risk reported by Jensen et al. was influenced by factors such as higher age at first birth among infertile women. Most previous studies have presented SIRs adjusted only for age and calendar year, which could explain why the risk of breast cancer was found higher in some of them [15, 17, 28–30]. It is reasonable to assume that parity and age at first birth act as mediators rather than confounders in the studied association. They can also be seen as a proxy for several different exposures, mainly changes in the tissue that occur during pregnancy and lactation that influence breast cancer risk. However, we found no associations with breast cancer risk whether adjusting for parity and age at first birth or not.

### Ovarian cancer

Infertile women had a higher incidence rate of ovarian cancer in our study. These results are similar to those of a large prospective cohort study [16], as well as two studies on infertile populations [14, 15]. In contrast, no increased risk was found in two case–control studies [31, 32] and two smaller studies involving infertile women only [17, 23]. Like most previous studies [27, 30, 33–36], we found a higher risk of ovarian cancer among women diagnosed with endometriosis. The association was similar in nulliparous and parous women, and also seemed to persist after menopause, though less pronounced. The mechanism behind this association is not entirely understood and several hypotheses have been proposed, including an increased oxidative stress caused by retrograde menstruation as well as changes in the microenvironment that promotes carcinogenesis [12]. We also found a higher risk of ovarian cancer among women diagnosed with ovulatory disturbances, which appeared to be confined to nulliparous premenopausal women. Women diagnosed both with infertility and ovulatory disturbances did not have a significantly higher risk of ovarian cancer, although it should be noted that estimates were based on small numbers. Increased levels of androgens in PCOS could theoretically influence ovarian cancer risk [11]. Previous studies on women with PCOS [26, 37], and women with androgen excess or menstrual disorders [29], have not found statistically significant associations with ovarian cancer. However, these studies included few ovarian cancer cases.

### Endometrial cancer

We found a higher risk of endometrial cancer among infertile compared to fertile women. The risk was also higher among women diagnosed with ovulatory disturbances, especially in combination with infertility. This risk appeared to be higher among nulliparous women and only evident before menopause. Our results are in agreement with those from a pooled analysis by Yang et al. [13], as well as several previous studies comparing risks among infertile women and women with PCOS to the general population [17, 23, 26, 29, 37, 38]. The study by Jensen et al. [14] did not find a higher risk of endometrial cancer among infertile women. Similar to a recent report from a prospective cohort in the US [27], women with endometriosis did not have a higher risk of endometrial cancer in our study. A Danish cohort reported a higher SIR of endometrial cancer in women with endometriosis, adjusted only for age and calendar year [30]. Overall, our results do not suggest an independent effect of infertility on endometrial cancer risk, but rather that the risk among women with infertility is associated with ovulatory disturbances. It is likely that PCOS is underlying the association, since the main problem with PCOS is a hormonal imbalance with a high ovarian estrogen production without progesterone counteraction, which in turn is a known risk factor for endometrial cancer. These features typically lead to menstrual irregularities due to a chronic and prolonged endometrial stimulation and a later age of menopause onset [39].

### Strengths and limitations

To our knowledge, this is one of the largest cohort studies to date on infertility and gynecological cancers. By utilizing Swedish population-based registers with essentially complete follow-up, we were able to accurately identify incident cancers as well as control for many important confounding variables while eliminating the risk of recall bias. The estimated completeness of the SCR for solid tumors is over 95% [40]. Diagnoses of infertility ascertained by physicians were recorded in the NPR for the duration of study follow-up.

Some limitations need to be considered when interpreting our findings. The NPR does not cover primary health care, and specialist out-patient care has only been included since 2001, meaning that some women with infertility-related problems were likely classified as non-infertile. This could bias the results towards the null, although any such effect is likely small given the large comparison group of non-infertile women. Diagnoses of infertility from in-patient care may differ from those recorded in out-patient care, and may reflect different subsets of women prior to and after 2001. However, infertility was more commonly treated in in-patient care during the beginning of the study period. Diagnoses of ovulatory disturbances were mainly captured in out-patient care records, resulting in shorter follow-up and fewer cancer cases among women with these diagnoses. It is also likely that many women with infertility due to ovulatory disturbances were categorized as having only a diagnosis of infertility. Since the cause of infertility was not specified for most women, the reasons for the associations with infertility alone could not be investigated further. The categories of infertility combined with endometriosis or ovulatory disturbances could also include women with infertility due to other causes, potentially diluting the associations.

A woman’s age at menarche and menopause is not recorded in population registers. Early menarche has been associated with a higher risk of ovarian, endometrial and breast cancer [4–6]. Since infertility seems to be more common in women with late menarche [41], this could bias the results towards the null. Age at menopause should not confound associations between infertility and cancer as it occurs after the reproductive period. In order to investigate potential effect modification by menopausal status, we used the 50th birthday as a cut-off.

For this large cohort of women, we used information collected on infertility and cancer diagnoses. However, in the group of women with a diagnosis of infertility, information on received hormonal treatments was not available. Therefore, we were unable to investigate whether the associations were mediated by any treatment. It is reassuring that most recent studies and meta-analyses have not found increased cancer risks in women who have undergone hormonal fertility treatments [42]. In a recent British study, ovarian cancer risk was higher than expected among women treated with assisted reproductive technology [9]. Because the higher risk was limited to women with endometriosis or low parity, the authors suggested that the risk was likely due to these characteristics and not the treatment itself.

Finally, we did not have information on additional hormonal treatments such as oral contraceptives or hormonal replacement after menopause, nor on the women’s weight. Oral contraceptives are associated with a lower risk of ovarian and endometrial cancer, and could potentially influence the studied associations if infertile women use them less than other women. On the other hand, oral contraceptives are also currently being prescribed for the treatment of endometriosis and PCOS. It is unlikely that differential use of these drugs could explain the results of this study. Obesity is associated with both PCOS and with an increased risk of endometrial cancer. However, we were unable to investigate whether this association was mediated by weight in our study.

## Conclusions

The results from this large population-based study suggest that infertile women may have a higher risk of certain cancers, such as gynecological cancers, but not of breast cancer. Our data confirm findings of previous studies on the increased risk of ovarian cancer in women with infertility and endometriosis, and support that ovulatory disturbances, probably due to underlying PCOS, increased the risk of endometrial cancer in infertile women. Additionally, we found an increased risk of ovarian cancer among nulliparous women with ovulatory disturbances. Women with endometriosis did not have a higher risk of endometrial cancer. Other large-scale studies are needed to confirm these findings.

While the treatment of infertility rightly focuses on helping the couple conceive, the long-term consequences of female infertility and related disorders also merit the attention of physicians and public health workers.

## Electronic supplementary material

Below is the link to the electronic supplementary material.

Supplementary material 1 (DOCX 30 kb)
