# Supplementary material for: The risk of breast and gynecological cancer in women with a diagnosis of infertility: a nationwide population-based study
Source: Eur J Epidemiol. 2019 Jan 9;34(5):499–507. doi: 10.1007/s10654-018-0474-9 (PMC6456460; doi:10.1007/s10654-018-0474-9)
Supplement: Supplementary file 1 — Supplementary material 1 (DOCX 30 kb) [file 10654_2018_474_MOESM1_ESM.docx]

**Supplementary table 1.** Diagnoses related to infertility in the International Classification of Diseases (ICD).

| **Diagnosis** | **ICD 7** | **ICD 8** | **ICD 9** | **ICD 10** |
| --- | --- | --- | --- | --- |
| Female infertility | 636 | 628 | 628 | N97 |
| Due to anovulation | N/A | N/A | 628A | N97.0 |
| Due to endometriosis | N/A | N/A | N/A | N97.8D |
| Endometriosis | N/A | 625,3 | 617 | N80 |
| Ovarian dysfunction | 275 | 256 | 256 | E28 |
| Absent, scanty or rare menstruation | 634,10-634,12 | 626,00-626,11 | 626A-626B | N91.1-N91.5 |

Note: In the Swedish counties ICD-7 was used before 1969, ICD-8 1969-1986, ICD-9 1987-1996 and ICD-10 from 1997. Skåne county switched to ICD-10 in 1998. N/A indicates that the diagnosis was not available in the current version of ICD.

**Supplementary table 2.** Associations between infertility and breast, ovarian and endometrial cancer, multivariable adjusted models without parity and age at first birth

| **Infertility** | **Breast cancer^a^** | | **Ovarian cancer^b^** | | **Uterine cancer^c^** | | |  |
| --- | --- | --- | --- | --- | --- | --- | --- | --- |
|  | **HR**  **(95% CI)** | ***p* value** | **HR**  **(95% CI)** | ***p* value** | **HR**  **(95% CI)** | ***p* value** |  |  |
| No infertility | 1.00 (reference) | . | 1.00 (reference) | . | 1.00 (reference) | . |  |  |
| Infertility | 1.03 (0.99-1.08) | 0.146 | 1.83 (1.65-2.03) | <0.001 | 1.59 (1.42-1.78) | <0.001 |  |  |
| **Infertility and related diagnoses** | | | | | | | | |
| No diagnosis | 1.00 (reference) | . | 1.00 reference) | . | 1.00 reference) | . |  |  |
| Ovulatory disturbances | 0.96 (0.86-1.07) | 0.426 | 1.61 (1.24-2.09) | <0.001 | 1.55 (1.19-2.01) | 0.001 |  |  |
| Endometriosis | 1.03 (0.97-1.08) | 0.367 | 1.86 (1.60-2.15) | <0.001 | 1.03 (0.83-1.28) | 0.768 |  |  |
| Infertility | 1.04 (0.99-1.09) | 0.087 | 1.82 (1.61-2.04) | <0.001 | 1.54 (1.36-1.75) | <0.001 |  |  |
| Infertility and ovulatory disturbances | 0.95 (0.79-1.14) | 0.570 | 1.27 (0.77-2.11) | 0.352 | 3.48 (2.47-4.90) | <0.001 |  |  |
| Infertility and endometriosis | 1.02 (0.90-1.15) | 0.805 | 2.72 (2.11-3.50) | <0.001 | 1.19 (0.80-1.78) | 0.398 |  |  |

^a^Adjusted for age, calendar time, education level, country of birth, salpingectomy, hysterectomy and bilateral oophorectomy.

^b^Adjusted for age, calendar time, education level, country of birth, salpingectomy and hysterectomy.

^c^Adjusted for age, calendar time, education level, country of birth, salpingectomy and bilateral oophorectomy.

**Supplementary table 3.** Associations between infertility and breast cancer by parity and by age

| **Infertility and related diagnoses** | **Cancer cases** | **Person-years** | **Age-adjusted** | | **Multivariable^a,b^** | |
| --- | --- | --- | --- | --- | --- | --- |
|  |  |  | **HR (95% CI)** | ***p* value** | **HR (95% CI)** | ***p* value** |
| **Nulliparous women** |  |  |  | |  | |
| No diagnosis | 6,760 | 21,543,299 | 1.00 (reference) | . | 1.00 (reference) | . |
| Ovulatory disturbances | 54 | 152,015 | 0.72 (0.55-0.94) | 0.015 | 0.71 (0.54-0.93) | 0.012 |
| Endometriosis | 237 | 180,455 | 0.98 (0.86-1.11) | 0.730 | 0.99 (0.87-1.13) | 0.881 |
| Infertility | 631 | 523,168 | 1.00 (0.92-1.08) | 0.988 | 1.00 (0.93-1.09) | 0.905 |
| Infertility and ovulatory disturbances | 38 | 56,090 | 0.92 (0.67-1.26) | 0.605 | 0.90 (0.66-1.24) | 0.529 |
| Infertility and endometriosis | 118 | 87,292 | 0.95 (0.80-1.14) | 0.612 | 0.96 (0.80-1.15) | 0.643 |
| **Parous women** |  |  |  |  |  |  |
| No diagnosis | 43,062 | 41,956,715 | 1.00 (reference) | . | 1.00 (reference) | . |
| Ovulatory disturbances | 276 | 222,971 | 1.03 (0.91-1.16) | 0.647 | 1.01 (0.90-1.14) | 0.808 |
| Endometriosis | 1,186 | 647,479 | 1.00 (0.95-1.06) | 0.894 | 1.03 (0.97-1.09) | 0.387 |
| Infertility | 1,102 | 881,570 | 1.04 (0.98-1.10) | 0.224 | 1.02 (0.96-1.09) | 0.478 |
| Infertility and ovulatory disturbances | 76 | 97,544 | 0.98 (0.78-1.23) | 0.859 | 0.94 (0.75-1.18) | 0.595 |
| Infertility and endometriosis | 147 | 114,221 | 1.02 (0.87-1.20) | 0.817 | 1.00 (0.85-1.17) | 0.971 |
| **Below age 50** |  |  |  | |  | |
| No diagnosis | 22,323 | 53,780,618 | 1.00 (reference) | . | 1.00 (reference) | . |
| Ovulatory disturbances | 131 | 306,627 | 0.88 (0.74-1.05) | 0.155 | 0.85 (0.71-1.00) | 0.056 |
| Endometriosis | 442 | 484,910 | 1.02 (0.93-1.12) | 0.653 | 1.02 (0.92-1.12) | 0.728 |
| Infertility | 778 | 1,091,451 | 1.00 (0.93-1.07) | 0.934 | 0.92 (0.86-0.99) | 0.022 |
| Infertility and ovulatory disturbances | 69 | 137,090 | 0.98 (0.77-1.24) | 0.861 | 0.87 (0.68-1.10) | 0.231 |
| Infertility and endometriosis | 106 | 151,735 | 0.88 (0.73-1.06) | 0.180 | 0.79 (0.65-0.95) | 0.015 |
| **Age 50 and older** |  |  |  |  |  |  |
| No diagnosis | 27,499 | 9,719,396 | 1.00 (reference) | . | 1.00 (reference) | . |
| Ovulatory disturbances | 199 | 68,359 | 1.04 (0.90-1.19) | 0.593 | 1.02 (0.88-1.17) | 0.810 |
| Endometriosis | 981 | 343,023 | 1.00 (0.94-1.06) | 0.976 | 1.02 (0.95-1.09) | 0.598 |
| Infertility | 955 | 313,287 | 1.11 (1.04-1.18) | 0.001 | 1.03 (0.96-1.09) | 0.450 |
| Infertility and ovulatory disturbances | 45 | 16,545 | 0.99 (0.74-1.33) | 0.959 | 0.92 (0.69-1.24) | 0.590 |
| Infertility and endometriosis | 159 | 49,778 | 1.17 (1.00-1.37) | 0.046 | 1.07 (0.91-1.25) | 0.414 |

^a^Adjusted for age, calendar time, education level, country of birth, salpingectomy, hysterectomy and bilateral oophorectomy.

^b^Model stratified by age also adjusted for parity and age at first birth.

Likelihood ratio test for effect modification by parity; age-adjusted p=0.2094, multivariable adjusted p=0.2580

Likelihood ratio test for effect modification by age; age-adjusted p=0.0313, multivariable adjusted p=0.0202

**Supplementary table 4.** Associations between infertility and ovarian cancer by parity and by age

| **Infertility and related diagnoses** | **Cancer cases** | **Person-years** | **Age-adjusted** | | **Multivariable^a,b^** | |
| --- | --- | --- | --- | --- | --- | --- |
|  |  |  | **HR (95% CI)** | ***p* value** | **HR (95% CI)** | ***p* value** |
| **Nulliparous women** |  |  |  | |  | |
| No diagnosis | 1,385 | 21,522,649 | 1.00 (reference) | . | 1.00 (reference) | . |
| Ovulatory disturbances | 25 | 151,012 | 1.87 (1.26-2.78) | 0.002 | 1.97 (1.32-2.92) | <0.001 |
| Endometriosis | 63 | 169,518 | 1.90 (1.48-2.45) | <0.001 | 2.20 (1.70-2.84) | <0.001 |
| Infertility | 136 | 518,283 | 1.42 (1.19-1.70) | <0.001 | 1.48 (1.23-1.76) | <0.001 |
| Infertility and ovulatory disturbances | 11 | 55,677 | 1.65 (0.91-2.99) | 0.097 | 1.79 (0.99-3.24) | 0.055 |
| Infertility and endometriosis | 31 | 83,506 | 1.79 (1.25-2.55) | 0.001 | 1.98 (1.39-2.84) | <0.001 |
| **Parous women** |  |  |  |  |  |  |
| No diagnosis | 4,408 | 41,818,262 | 1.00 (reference) | . | 1.00 (reference) | . |
| Ovulatory disturbances | 33 | 220,244 | 1.24 (0.88-1.75) | 0.217 | 1.31 (0.93-1.84) | 0.123 |
| Endometriosis | 146 | 598,160 | 1.39 (1.18-1.64) | <0.001 | 1.66 (1.40-1.98) | <0.001 |
| Infertility | 163 | 874,929 | 1.55 (1.33-1.82) | <0.001 | 1.66 (1.42-1.94) | <0.001 |
| Infertility and ovulatory disturbances | 4 | 96,720 | 0.50 (0.19-1.34) | 0.169 | 0.56 (0.21-1.50) | 0.251 |
| Infertility and endometriosis | 30 | 110,577 | 2.22 (1.55-3.18) | <0.001 | 2.57 (1.80-3.69) | <0.001 |
| **Below age 50** |  |  |  | |  | |
| No diagnosis | 2,826 | 53,750,460 | 1.00 (reference) | . | 1.00 (reference) | . |
| Ovulatory disturbances | 39 | 305,204 | 2.20 (1.61-3.02) | <0.001 | 2.20 (1.61-3.02) | <0.001 |
| Endometriosis | 86 | 468,105 | 2.06 (1.66-2.56) | <0.001 | 2.20 (1.77-2.74) | <0.001 |
| Infertility | 157 | 1,087,329 | 1.90 (1.62-2.23) | <0.001 | 1.71 (1.45-2.01) | <0.001 |
| Infertility and ovulatory disturbances | 11 | 136,517 | 1.34 (0.74-2.42) | 0.333 | 1.36 (0.75-2.46) | 0.308 |
| Infertility and endometriosis | 38 | 148,744 | 3.13 (2.27-4.31) | <0.001 | 2.89 (2.09-3.99) | <0.001 |
| **Age 50 and older** |  |  |  |  |  |  |
| No diagnosis | 2,967 | 9,590,451 | 1.00 (reference) | . | 1.00 (reference) | . |
| Ovulatory disturbances | 19 | 66,052 | 0.94 (0.60-1.47) | 0.782 | 0.94 (0.60-1.47) | 0.771 |
| Endometriosis | 123 | 299,574 | 1.31 (1.10-1.57) | 0.003 | 1.53 (1.27-1.85) | <0.001 |
| Infertility | 142 | 305,883 | 1.54 (1.31-1.83) | <0.001 | 1.37 (1.15-1.62) | <0.001 |
| Infertility and ovulatory disturbances | 4 | 15,880 | 0.84 (0.32-2.24) | 0.730 | 0.77 (0.29-2.06) | 0.603 |
| Infertility and endometriosis | 23 | 45,340 | 1.71 (1.13-2.57) | 0.011 | 1.55 (1.03-2.35) | 0.036 |

^a^Adjusted for age, calendar time, education level, country of birth, salpingectomy and hysterectomy.

^b^Model stratified by age also adjusted for parity and age at first birth.

Likelihood ratio test for effect modification by parity; age-adjusted p=0.0299, multivariable adjusted p=0.0333

Likelihood ratio test for effect modification by age; age-adjusted p=0.0001, multivariable adjusted p=0.0002

**Supplementary table 5.** Associations between infertility and endometrial cancer by parity and by age

| **Infertility and related diagnoses** | **Cancer cases** | **Person-years** | **Age-adjusted** | | **Multivariable^a,b^** | |
| --- | --- | --- | --- | --- | --- | --- |
|  |  |  | **HR (95% CI)** | ***p* value** | **HR (95% CI)** | ***p* value** |
| **Nulliparous women** |  |  |  | |  | |
| No diagnosis | 1,190 | 21,424,043 | 1.00 (reference) | . | 1.00 (reference) | . |
| Ovulatory disturbances | 28 | 150,310 | 2.15 (1.48-3.13) | <0.001 | 2.20 (1.51-3.20) | <0.001 |
| Endometriosis | 26 | 146,135 | 0.83 (0.56-1.22) | 0.345 | 0.87 (0.59-1.28) | 0.469 |
| Infertility | 143 | 502,266 | 1.26 (1.06-1.50) | 0.008 | 1.28 (1.08-1.53) | 0.005 |
| Infertility and ovulatory disturbances | 20 | 54,695 | 3.36 (2.16-5.23) | <0.001 | 3.46 (2.22-5.38) | <0.001 |
| Infertility and endometriosis | 19 | 74,998 | 1.06 (0.67-1.67) | 0.796 | 1.10 (0.70-1.73) | 0.681 |
| **Parous women** |  |  |  |  |  |  |
| No diagnosis | 4,461 | 40,956,804 | 1.00 (reference) | . | 1.00 (reference) | . |
| Ovulatory disturbances | 29 | 212,767 | 1.06 (0.74-1.53) | 0.751 | 1.09 (0.75-1.57) | 0.656 |
| Endometriosis | 63 | 375,934 | 0.98 (0.76-1.25) | 0.859 | 1.01 (0.79-1.30) | 0.934 |
| Infertility | 115 | 849,462 | 1.15 (0.96-1.39) | 0.128 | 1.19 (0.98-1.43) | 0.072 |
| Infertility and ovulatory disturbances | 13 | 95,230 | 2.17 (1.26-3.73) | 0.005 | 2.28 (1.32-3.93) | 0.003 |
| Infertility and endometriosis | 5 | 99,758 | 0.48 (0.20-1.15) | 0.098 | 0.51 (0.21-1.22) | 0.128 |
| **Below age 50** |  |  |  | |  | |
| No diagnosis | 953 | 53,393,925 | 1.00 (reference) | . | 1.00 (reference) | . |
| Ovulatory disturbances | 27 | 301,816 | 4.28 (2.92-6.28) | <0.001 | 4.09 (2.79-5.99) | <0.001 |
| Endometriosis | 16 | 374,581 | 1.26 (0.77-2.06) | 0.364 | 1.17 (0.71-1.92) | 0.537 |
| Infertility | 66 | 1,069,762 | 2.00 (1.55-2.56) | <0.001 | 1.66 (1.29-2.13) | <0.001 |
| Infertility and ovulatory disturbances | 24 | 135,334 | 8.63 (5.75-12.94) | <0.001 | 8.09 (5.38-12.15) | <0.001 |
| Infertility and endometriosis | 6 | 139,653 | 1.30 (0.58-2.90) | 0.523 | 1.07 (0.48-2.40) | 0.862 |
| **Age 50 and older** |  |  |  |  |  |  |
| No diagnosis | 4,698 | 8,986,922 | 1.00 (reference) | . | 1.00 (reference) | . |
| Ovulatory disturbances | 30 | 61,261 | 0.96 (0.67-1.37) | 0.818 | 0.92 (0.64-1.31) | 0.636 |
| Endometriosis | 73 | 147,488 | 0.96 (0.76-1.21) | 0.713 | 0.90 (0.71-1.14) | 0.387 |
| Infertility | 192 | 281,966 | 1.39 (1.21-1.61) | <0.001 | 1.11 (0.96-1.29) | 0.166 |
| Infertility and ovulatory disturbances | 9 | 14,591 | 1.28 (0.66-2.46) | 0.463 | 1.08 (0.56-2.08) | 0.821 |
| Infertility and endometriosis | 18 | 35,103 | 1.09 (0.69-1.74) | 0.704 | 0.82 (0.52-1.31) | 0.409 |

^a^Adjusted for age, calendar time, education level, country of birth, salpingectomy and bilateral oophorectomy.

^b^Model stratified by age also adjusted for parity and age at first birth.

Likelihood ratio test for effect modification by parity; age-adjusted p=0.0337, multivariable adjusted p=0.0414

Likelihood ratio test for effect modification by age; age-adjusted p<0.0001, multivariable adjusted p<0.0001
